# Evaluating antibiotic regimens for streptococcal toxic shock syndrome in children

**DOI:** 10.1371/journal.pone.0292311

**Published:** 2023-10-12

**Authors:** Haixia Zhang, Jie Dong, Jiaotian Huang, Keyuan Zhang, Xiulan Lu, Xin Zhao, Changqiong Xiao

**Affiliations:** 1 Department of Pharmacy, Hunan Children’s Hospital, Changsha, China; 2 Pediatrics Research Institute of Hunan Province, Hunan Children’s Hospital, Changsha, China; 3 Department of Intensive Care Medicine, Hunan Children’s Hospital, Changsha, China; 4 School of Medicine, Hunan Normal University, Changsha, China; 5 Department of Pharmacy, Chenzhou First People’s Hospital, Chenzhou, China; Aga Khan University Hospital, PAKISTAN

## Abstract

**Introduction:**

Streptococcal toxic shock syndrome (STSS) is a severe consequence of infections from Streptococcus pyogenes. The early identification and timely intervention with appropriate anti-infective agents are pivotal for managing pediatric STSS. This study evaluates the effectiveness of various treatment regimens for STSS in children.

**Methods:**

Clinical data of children with STSS resulting from β-hemolytic streptococcal infections in two hospitals were retrospectively analyzed from January 2009 to April 2023. Additionally, literature from the China National Knowledge Infrastructure on pediatric STSS was examined. Antimicrobial treatments were categorized into four groups based on their compositions, with an additional categorization for adjunct therapeutic drugs.

**Results:**

Of 32 confirmed STSS cases, all displayed sensitivity to ampicillin, β-lactam antibiotics, and vancomycin, but resistance to clindamycin, erythromycin, and tetracycline. From the literature, 23 studies with 50 cases were extracted, leading to a total of 82 patients for evaluation. The efficacy rates varied significantly among the four treatment groups. Notably, the standard penicillin-containing group exhibited the highest efficacy (86.4%), while the group with macrolides/unused antibiotics registered a 0% efficacy rate. The other two groups demonstrated efficacy rates of 32.1% and 42.3%.

**Conclusion:**

For pediatric STSS, Streptococcus pyogenes shows notable sensitivity to ampicillin. Implementing timely β-lactam antibiotics, specifically penicillin, in conjunction with clindamycin and intravenous immunoglobulins enhances the treatment success rate.

## Introduction

Streptococcal toxic shock syndrome (STSS) represents a rare yet profoundly serious clinical infection. It manifests with rapid progression and has the potential to culminate in shock, multi-organ failure, and significant mortality within a limited timeframe. For pediatric patients diagnosed with STSS, early detection paired with swift and comprehensive anti-infective and symptomatic intervention becomes pivotal. Despite the severity and complexity of this syndrome, research focused on pediatric STSS within the Chinese medical landscape remains scant. The majority of available literature is predominantly composed of isolated case reports, leaving a noticeable gap in comprehensive studies, especially those scrutinizing drug efficacy. The landscape of clinical anti-infective and adjunct drug regimens currently suffer from a lack of standardization. As a result, clinicians often lean towards deploying either broad-spectrum or ultra-broad-spectrum antibacterial agents [1–3]. In light of this, our research embarked on retrospective exploration of pediatric STSS cases spanning two prominent hospitals over the previous decade. Our primary objective was to juxtapose and critically analyze the outcomes stemming from diverse drug treatment regimens, with an overarching aim of enhancing the rate of successful treatment interventions.

## Materials and methods

### Search strategy and selection criteria

This was a retrospective analysis of cases and literature conducted on April 30, 2023. The test query systems of Hunan Children’s Hospital and First People’s Hospital of Chenzhou were searched for children with bacterial culture results of Streptococcus pyogenes in sterile specimens from January 2009 to April 2023. Participant data were collected in the same date range and anonymized to ensure patient confidentiality. Data collection was approved by the Medical Ethics Committee of Hunan Children’s Hospital (HCHLL-2023-39) and the First People’s Hospital of Chenzhou (2023104), and the need for informed consent was waived. This study was performed in accordance with the Declaration of Helsinki of 1964 and its subsequent amendments. All data retrieved from clinics as part of the audit were anonymized, and no informed consent was required from the participants for study enrolment or publication purposes.

The diagnostic criteria for STSS were as follows: (1) isolation of Streptococcus pyogenes from a sterile site in the patient, (2) hypotension (systolic blood pressure <5th percentile for age), and (3) multiorgan involvement characterized by two or more of the following:

1. Renal impairment: ≥2 times the upper normal limit for age; ≥2 times elevation over the baseline in patients with pre-existing renal disease
2. Coagulopathy: platelets ≤100 × 10^9^/L or disseminated intravascular coagulation defined by prolonged clotting times, low fibrinogen levels, and the presence of fibrin degradation products
3. Liver involvement: alanine aminotransferase, aspartate aminotransferase, or total bilirubin levels ≥2 times the upper normal limit for the patient’s age; ≥2 times elevation over the baseline in patients with pre-existing liver disease
4. Acute respiratory distress syndrome
5. Erythematous macular rash, may desquamate
6. Soft tissue necrosis (e.g., necrotizing fasciitis, myositis, or gangrene) [4, 5].

The inclusion criteria were as follows: (1) aged <18 years, (2) meeting the diagnostic criteria for STSS, (3) underwent hospitalization for medication administration, and (4) having detailed medical records. The case exclusion criteria were as follows: (1) aged ≥18 years; (2) receiving other drugs or adjuvant treatment during the study that significantly affected the outcome of the trial; (3) having a malignancy; and (4) having incomplete case data.

### Identification of streptococcus and treatment regimen grouping

Streptococci were identified by their morphological growth using blood plates, beta hemolysis, and Gram staining, followed by automated bacterial identification systems. The findings were interpreted according to the guidelines of the Clinical and Laboratory Standards Institute (CLSI) [6].

General information, clinical presentation, test results, treatment drugs, efficacy, and adverse effects were collected for all included cases. The antimicrobial treatment regimens were divided into four groups based on consensus and relevant literature [5, 7–10]: penicillin-containing regimen (standard group); carbapenem+glycopeptides/linezolid (Group A); carbapenem, broad-spectrum antibiotics, and glycopeptides/linezolid alone or in combination, except for Group A (Group B); and macrolides/unused antibiotics (Group C). Other therapeutic drugs were divided into intravenous immunoglobulins (IVIG) and glucocorticoids.

### Literature search strategy and literature screening

Databases such as "China Knowledge Network,” "Wanfang," and "Wipu" were selected, and a combination of subject terms and free words were used in the search strategy. "Streptococcal toxic shock syndrome,” "streptococcus,” "toxic shock syndrome,” "shock,” "septic toxicity," and "children" were used as search terms, and the time frame was set from January 1981 to April 2023. Two researchers independently read the literature and screened the studies according to the diagnostic and exclusion criteria. A third-party consultation was performed in cases of disagreement between the two.

Data were extracted from the final included studies, which included authors, time of publication, number of cases, basic characteristics of the study population (sex and age), interventions, and outcome indicators. Two evaluators screened the literature, independently extracted the data, and cross-checked them, and a third party was consulted in case of disagreements to reach a consensus. Literature quality was evaluated using the Cochrane Collaboration Network Risk-of-Bias Assessment Tool [11].

### Evaluation of clinical efficacy

The clinical efficacy of the antimicrobial drugs was evaluated 3–7 days after treatment initiation and at the end of the treatment course according to the Technical Guidelines for Clinical Trials of Antimicrobial Drugs, as follows [12]: (i) effective: the patient’s symptoms and signs disappeared or recovered completely after drug discontinuation, and laboratory tests returned to normal; (ii) invalid: the patient’s symptoms and signs persisted, did not completely disappear, or even deteriorated after drug discontinuation, or new symptoms and signs appeared and/or new antimicrobial treatment measures were used.

### Data analysis

Statistical analysis was performed using SPSS software version 23.0, and the median (interquartile range) was used for data measurement. The Mann–Whitney U test was used for comparison between two groups, and the Wilcoxon test for paired samples was used to compare the inflammatory indices before and after treatment. Statistical data were expressed as n (%), and differences between groups were analyzed using the χ^2^ test or Fisher’s exact text. Bilateral *P*<0.05 was considered a statistically significant difference.

## Results

### Demographic characteristics

A total of 32 children were diagnosed with STSS during the study period (15 boys, 17 girls). The median (interquartile range) age and weight were 3 (1,53) months and 4.9 (3.7,19) kg, respectively. Eleven cases had underlying diseases, including three with chickenpox, one with lymphoma of the right lower limb, one with submaxillary abscess, one with cellulitis of the lower back, one with a neck mass, one with scalp trauma, one with precocious heart disease, one with a very low birth weight preterm infant at 29^+2^ weeks of gestation, and one with developmental delay, with no underlying diseases in the remaining 21 cases. The median (interquartile range) time between symptom onset and hospitalization was 3(1,5) days. The median (interquartile range) duration of hospitalization was 15(5,29) days. Nine patients (28.1%) were cured, 10 (31.3%) were discharged on improvement, 12 (37.5%) died, and one (3.1%) dropped out of follow-up.

### Clinical characteristics

Ten (31.3%) children had normal body temperature, one (3.1%) was premature, and one (3.1%) was hypothermic at age 1 month. The remaining 20 (63%) children had a fever, and the febrile children had a median temperature of 39.1 (38.8,39.6) °C on admission. The primary focus of infection was the respiratory tract in 13 cases (40.6%), soft tissues of the skin in eight cases (25%), central nervous system in seven cases (21.9%), and bloodstream and gastrointestinal tract in two cases (6.3%). Six (18.8%) patients developed skin rashes. The organ systems involved included the lungs, heart, nervous system, liver, kidneys, gastrointestinal tract, coagulation system, and skin. Four cases (12.5%) involved six sites simultaneously, seven cases (21.9%) involved five sites, four cases (12.5%) involved four sites, nine cases (28.1%) involved three sites, and eight cases (25%) involved two sites.

### Pathogenic tests and drug sensitivity tests

Cultures of sterile specimens from all 32 children showed growth of Streptococcus pyogenes, including 22 blood specimens (68.8%), of which two cerebrospinal fluid specimens were also found to contain the same organism, three cases each of pleural fluid, ascites, and intraoperative biopsy tissue (9.4%), and one case of cerebrospinal fluid (3.1%). All strains were sensitive to β-lactam antibacterial drugs, such as ampicillin and vancomycin, and were resistant to clindamycin, erythromycin, and tetracycline.

### Medication regimen and efficacy

The standard group was the most common antimicrobial regimen among the 32 children (11/32, 34.4%), with 19 (59.4%) treated with IVIG and 10 (31.3%) treated with glucocorticoids. There was a significant difference in the efficacy rates among the different antimicrobial regimens (P < 0.05). The efficiency rate was the highest in the standard group. There was no significant difference in the efficacy of other drug treatment regimens, including IVIG and glucocorticoids (P>0.05).

A total of 23 papers with 50 cases were included in the stratified screening process. Of these, 28 were boys and 22 were girls. The age range of the children was 5 h–18 years, and the length of stay was 2 h–61 days. Ten patients (20%) were cured, 10 (20%) improved, 28 (56%) died, and two (4%) dropped out of follow-up. Streptococcus pyogenes was detected in 50 cases, with the vast majority of cases being susceptible to β-lactams, such as ampicillin and vancomycin, and resistant to clindamycin, erythromycin, and tetracycline. Twenty-nine patients (58%) were treated with IVIG, and 13 (26%) were treated with glucocorticoids. Group A was the most common antimicrobial drug regimen (36% of 50 patients), and there was a significant difference in the efficacy rates of different antimicrobial drug regimens (*P*<0.05). The standard group exhibited the highest efficacy (72.7%).

A total of 82 cases were pooled from the 32 cases in our study and 50 cases reported in the literature. After increasing the sample size to 82 cases, group A remained the most common antimicrobial regimen (34.1%), followed by group B (31.7%), the standard group (26.8%), and group C (7.3%). There was a significant difference in the efficacy rates of the four regimens (*P*<0.001), with the standard group having the highest rate at 86.4%, group C having the lowest at 0, and groups A and B having rates of 32.1% and 42.3%, respectively.

**Fig 1:**
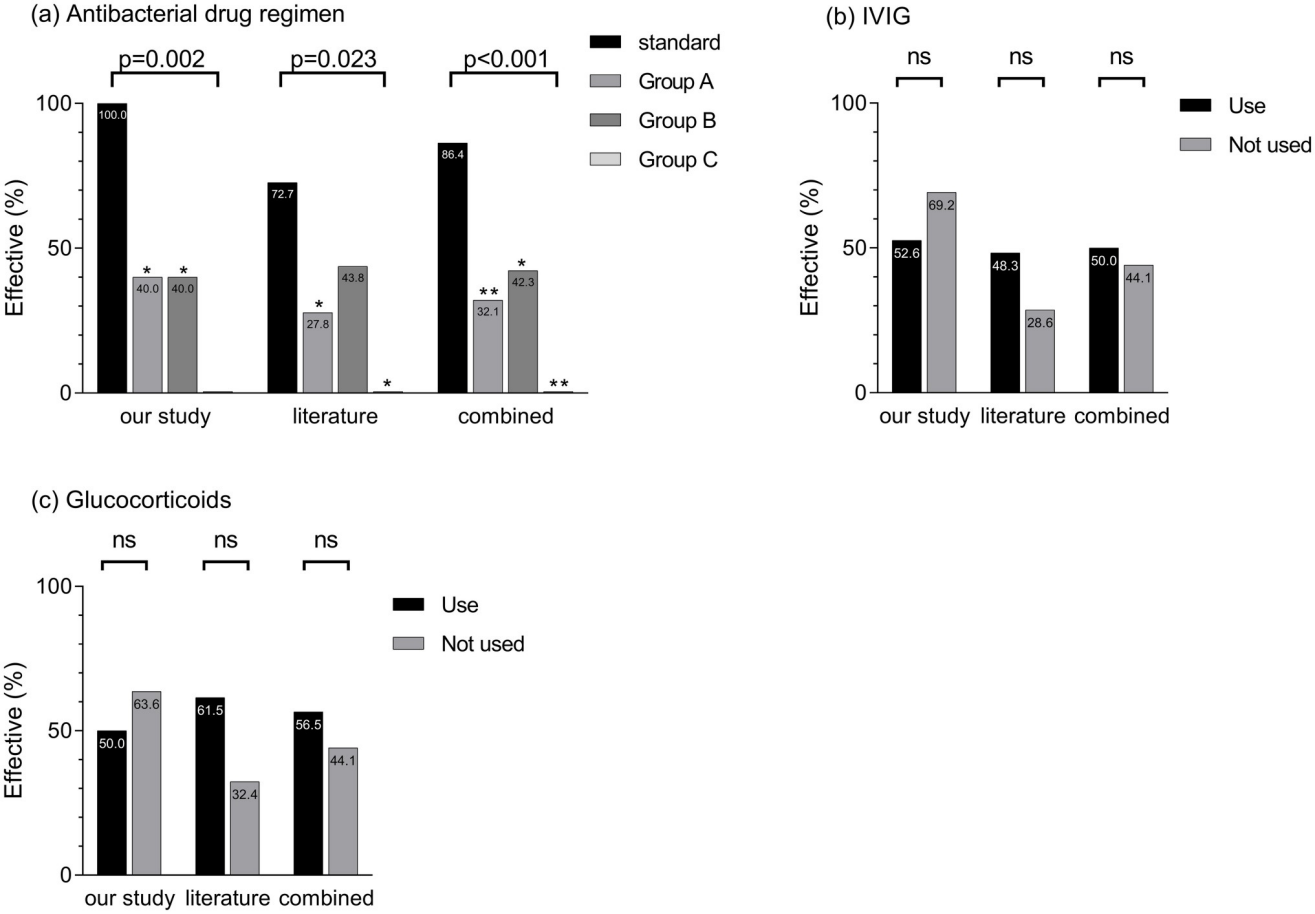
Comparison of the efficacy of drug treatment regimens in children with STSS. Standard group: penicillin ± other antibiotics. Group A: carbapenems + glycopeptides/linezolid. Group B: Carbapenems, broad-spectrum antibiotics, and glycopeptides/linezolid alone or in combination, except for Group A. Group C: macrolides/unused antibiotics. Effective: The patient’s signs and symptoms disappeared or recovered completely after drug discontinuation, and laboratory test results returned to normal. Invalid: the patient’s symptoms and signs persisted, did not disappear completely, or even deteriorated after drug discontinuation, or new symptoms and signs appeared and/or new antimicrobial treatment measures were used. *P<0.05, **P<0.001.

Specific antimicrobial and other drug treatment regimens and their efficacy comparisons are shown in Fig 1.

## Discussion

STSS in children has not been widely reported in China; however, its incidence has increased in recent years [13–15]. Hua et al. [1] collected 15 cases of STSS in children from seven hospitals in China over 8 years, mainly with soft-tissue skin and lung infections; the most common empirical regimen was that of carbapenems combined with glycopeptides or linezolid. We collected 32 cases of confirmed STSS over 14 years, mainly distributed over the last 8 years, which also indicates the increasing trend of STSS incidence. The increased detection rate of the disease can be attributed to microbial equipment quality improvement, enhancement of clinical diagnostic awareness of the disease, the environment, living standards, the COVID-19 pandemic leading to "immune debt," and other factors. In this study, the primary sites of infection were also most frequent in the respiratory tract, skin, and soft tissues, suggesting that these sites of infection are the most likely sources for the development of invasive infections and clinical attention should be given to skin and soft tissue infections in children. All 15 cases of STSS in children reported by Hua et al. had high fevers. In contrast, we had 12 children with normal body temperature or hypothermia, most of whom were young. The early manifestations of infection in neonates and younger infants are often atypical, and these children may be febrile or have normal temperatures; therefore, it is necessary to be alert to the progression of the infection to STSS in younger infants and children with hypothermia and normal body temperatures.

Our study showed that a treatment regimen of carbapenems combined with glycopeptides or linezolid and broad-spectrum antibiotics was common; however, the efficacy rate of this regimen was lower than that of the penicillin-containing group. β-lactams and vancomycin are bactericidal agents that inhibit bacterial cell wall synthesis, and they differ in their target sites of action. Furthermore, β-lactams have a faster anticolonic effect than vancomycin, and penicillin or ampicillin has the best activity and narrowest antibacterial spectrum against Streptococcus pyogenes. Epidemiological studies show that Streptococcus pyogenes is highly sensitive to penicillin [6, 16, 17], and that penicillin is the best microbicide choice for Streptococcus pyogenes caused-STSS [9, 10]. Streptococcus pyogenes is highly resistant to erythromycin and clindamycin. All patients in group C who used macrolides or did not use antibiotics had a poor prognosis. The clinical efficacy also suggests that the use of macrolides, such as erythromycin, should be avoided in STSS caused by Streptococcus pyogenes, even if the patient has a β-lactam allergy.

In addition to the early use of sensitive bactericides and antibiotics, clindamycin and IVIGs should be used as adjuncts for STSS treatment. Clindamycin has a high drug resistance rate; however, it has better tissue permeability and a longer post-antibiotic effect than penicillin and can inhibit invasive enzymes and exotoxins, which reduces mortality. Therefore, clindamycin is recommended for use in combination with other drugs [7, 10]. Only five patients were treated with clindamycin in this study. Therefore, it is necessary to improve pediatricians’ overall understanding of this disease, which is not limited to bacterial resistance. The efficacy rates of IVIGs and glucocorticoids did not show a significant difference; however, the efficacy rate of the standardized combination of clindamycin and IVIGs was high. IVIGs can neutralize toxins, inhibit inflammatory reactions, and improve treatment efficiency, whereas glucocorticoids are not recommended for STSS treatment [8–10].

The limitations of our study were the scarcity of cases, the lack of sufficient detail to facilitate stratification of illness severity in case information in the literature, such as the Sequential Organ Failure Assessment Score, and a large proportion of children dying or abandoning the treatment before the bacterial culture results report, without the opportunity to adjust the antimicrobials, which may have affected the efficacy rate.

## Conclusion

The results of this study indicated that Streptococcus pyogenes, which causes STSS in children, exhibits high sensitivity to ampicillin and high resistance to clindamycin and erythromycin. Macrolides should be avoided owing to their high mortality rates. Ultra-broad-spectrum and special-grade antibiotics are frequently used in this condition; however, their efficacy rates are lower than the penicillin-containing regimens. Standardization of the combination therapy with clindamycin and IVIGs is recommended to improve the treatment success rate. Clinicians should be concerned about the rapid progression of STSS and its associated high mortality rates.

## Supporting information

S1 File Minimal data. (ZIP)

## References

[1] Hua CZ, Yu H, Yang LH, Xu HM, Lyu Q, Lu HP, et al. Streptococcal toxic shock syndrome caused by Streptococcus pyogenes: a retrospective study of 15 pediatric cases. Zhonghua Er Ke Za Zhi. 2018;56: 587–591. doi: 10.3760/cma.j.issn.0578-1310.2018.08.006 30078239

[2] Lu WF, Lou ZX, Li J, Zeng DS. A case of streptococcal toxic shock syndrome mainly manifesting as multiple ecthyma gangrenosum. Zhonghua Pi Fu Ke Zhi. 2021;54: 729–730.

[3] Dong L, He SJ, Zhang YL, Zhou XC. Streptococcal toxic shock syndrome: report of 2 cases. Zhonghua Er Ke Za Zhi. 2007;45: 306–307. 17706074

[4] The working group on severe streptococcal infections. Defining the group A streptococcal toxic shock syndrome. Rationale and consensus definition. JAMA. 1993;269: 390–391. doi: 10.1001/jama.1993.03500030088038 8418347

[5] Centers for Disease Control and Prevention. Streptococcal toxic shock syndrome (STSS) (Streptococcus pyogenes). [Cited September 18, 2018]. https://ndc.services.cdc.gov/conditions/streptococcal-toxic-shock-syndrome/.

[6] CLSI performance standards for antimicrobial susceptibility testing. EDN. Wayne, Pennsylvania: Clinical and Laboratory Standards Institute; 2022, Volume M100. 32st.

[7] Parks T, Wilson C, Curtis N, Norrby-Teglund A, Sriskandan S. Polyspecific intravenous immunoglobulin in clindamycin-treated patients with streptococcal toxic shock syndrome: A systematic review and meta-analysis. Clin Infect Dis. 2018;67: 1434–1436. doi: 10.1093/cid/ciy401 29788397 PMC6186853

[8] Gottlieb M, Long B, Koyfman A. The evaluation and management of toxic shock syndrome in the emergency department: a review of the literature. J Emerg Med. 2018;54: 807–814. doi: 10.1016/j.jemermed.2017.12.048 29366615

[9] Babiker A, Kadri SS. ICU management of invasive β-hemolytic streptococcal infections. Infect Dis Clin North Am. 2022;36: 861–887. doi: 10.1016/j.idc.2022.07.007 36328640

[10] Group A streptococcal infections. Red. In: Kimberlin DW, Brady MT, Jackson MA, Long SS, editors. Report of the Committee on Infectious Diseases, 30th, Book 2015. Elk Grove Village, Illinois: American Academy of Pediatrics; 2015. p. 732.

[11] Cumpston M, LiT J, Page MJ, Chandler J, Welch VA, Higgins JP, et al. Updated guidance for trusted systematic reviews: a new edition of the Cochrane Handbook for Systematic Reviews of Interventions [J]. Cochrane Database Syst Rev. 2019;10: E000142. 10.1002/14651858.ED000142 PMC10284251 31643080

[12] Technical guidelines for clinical trials of antimicrobial drugs writing group. Technical guidelines for clinical trials of antimicrobial drugs. Chin J Clin Pharmacol. 2014;30: 844–856.

[13] van Kempen EB, Bruijning-Verhagen PCJ, Borensztajn D, Vermont CL, Quaak MSW, Janson JA, et al. Increase in invasive group a streptococcal infections in children in the Netherlands, A survey among 7 hospitals in 2022. Pediatr Infect Dis J. 2023;42: e122–e124. doi: 10.1097/INF.0000000000003810 36728741

[14] Dunne EM, Hutton S, Peterson E, Blackstock AJ, Hahn CG, Turner K, et al. Increasing incidence of invasive Group A streptococcus disease, Idaho, USA, 2008–2019. Emerg Infect Dis. 2022;28: 1785–1795. doi: 10.3201/eid2809.212129 35997313 PMC9423907

[15] Ladhani SN, Guy R, Bhopal SS, Brown CS, Lamagni T, Sharp A. Paediatric group A streptococcal disease in England from October to December, 2022. Lancet Child Adolesc Health. 2023;7: e2–e4. doi: 10.1016/S2352-4642(22)00374-1 36566755

[16] Yu D, Zheng Y, Yang Y. Is there emergence of β-lactam antibiotic-resistant *Streptococcus pyogenes* in China? Infect Drug Resist. 2020;13: 2323–2327. doi: 10.2147/IDR.S261975 32765008 PMC7369151

[17] Sun L, Xiao Y, Huang W, Lai J, Lyu J, Ye B, et al. Prevalence and identification of antibiotic-resistant scarlet fever group A Streptococcus strains in some paediatric cases at Shenzhen, China. J Glob Antimicrob Resist. 2022;30: 199–204. doi: 10.1016/j.jgar.2022.05.012 35618209

