# Supplementary material for: Evaluating antibiotic regimens for streptococcal toxic shock syndrome in children
Source: PLoS One. 2023 Oct 12;18(10):e0292311. doi: 10.1371/journal.pone.0292311 (PMC10569614; doi:10.1371/journal.pone.0292311)
Supplement: S1 File — (ZIP) [file pone.0292311.s001.zip › Supplementary files/minimal data.docx]

Table 1 Comparison of the efficacy of drug treatment regimens in 32 children with STSS

|  | Effective (%) | Invalid (%) | *P*-value |
| --- | --- | --- | --- |
| Antibacterial drug regimen |  |  | **0.002** |
| Standard group (penicillin ± other antibiotics) | 11 (100) | 0 (0) | - |
| Group A (carbapenems + glycopeptides/linezolid) | 4 (40.0) | 6 (60.0) | **0.004** |
| Group B (carbapenems, broad-spectrum antibiotics, glycopeptides/linezolid alone or in combination, except group A) | 4 (40.0) | 6 (60.0) | **0.004** |
| Group C (macrolides/no antimicrobial use) | 0 (0) | 1 (100) | 0.083 |
| IVIG |  |  | 0.348 |
| Use | 10（52.6） | 9（47.4） |  |
| Not used | 9（69.2） | 4（30.8） |  |
| Glucocorticoids |  |  | 0.699 |
| Use | 5 (50.0) | 5 (50.0) |  |
| Not used | 14 (63.6) | 8 (36.4) |  |
| Total | 19 (59.4) | 13 (40.6) |  |

Table 2 Comparison of the efficacy of drug treatment regimens in 50 children with STSS reported in the domestic literature

|  | Effective (%) | Invalid (%) | *χ^2^* | P-value |
| --- | --- | --- | --- | --- |
| Antibacterial drug regimen |  |  |  | **0.023** |
| Standard group (penicillin ± other antibiotics) | 8 (72.7) | 3 (27.3) |  | - |
| Group A (carbapenems + glycopeptides/linezolid) | 5 (27.8) | 13 (72.2) |  | **0.027** |
| Group B (carbapenems, broad-spectrum antibiotics, glycopeptides/linezolid alone or in combination, except group A) | 7 (43.8) | 9 (56.2) |  | 0.239 |
| Group C (macrolides/no antimicrobial use) | 0 (0) | 5 (100.0) |  | **0.026** |
| IVIG |  |  | 1.97 | 0.16 |
| Use | 14 (48.3) | 15 (51.7) |  |  |
| Not used | 6 (28.6) | 15 (71.4) |  |  |
| Glucocorticoids |  |  | 3.40 | 0.065 |
| Use | 8 (61.5) | 5 (38.5) |  |  |
| Not used | 12 (32.4) | 25 (67.6) |  |  |
| Total | 20 (40.0) | 30 (60.0) |  |  |

Table 3 Comparison of the efficacy of drug treatment regimens in 82 children with STSS

|  | Effective (%) | Invalid (%) | *χ^2^* | P-value |
| --- | --- | --- | --- | --- |
| Antibacterial drug regimen |  |  |  | **<0.001** |
| Standard group (penicillin ± other antibiotics) | 19 (86.4) | 3 (13.6) |  | - |
| Group A (carbapenems + glycopeptides/linezolid) | 9 (32.1) | 19 (67.9) | 14.70 | **<0.001** |
| Group B (carbapenems, broad-spectrum antibiotics, glycopeptides/linezolid alone or in combination, except group A) | 11 (42.3) | 15 (57.7) | 9.87 | **0.002** |
| Group C (macrolides/no antimicrobial use) | 0 (0) | 6 (100) |  | **<0.001** |
| IVIG |  |  | 0.28 | 0.599 |
| Use | 24 (50.0) | 24 (50.0) |  |  |
| Not used | 15 (44.1) | 19 (55.9) |  |  |
| Glucocorticoids |  |  | 1.03 | 0.31 |
| Use | 13 (56.5) | 10 (43.5) |  |  |
| Not used | 26 (44.1) | 33 (55.9) |  |  |
| Total | 39 (47.6) | 43 (52.4) |  |  |
